# Clever Cooperation: Interactions Between EspF and Host Proteins

**DOI:** 10.3389/fmicb.2018.02831

**Published:** 2018-11-22

**Authors:** Ying Hua, Kaina Yan, Chengsong Wan

**Affiliations:** ^1^Department of Microbiology, School of Public Health, Southern Medical University, Guangzhou, China; ^2^Key Laboratory of Tropical Disease Research of Guangdong Province, Guangzhou, China

**Keywords:** EPEC, EHEC (enterohaemorrhagic *E. coli*), EspF, protein interactions, bacterial pathogenesis

## Abstract

EspF is a central effector protein of enterohemorrhagic *Escherichia coli* (EHEC), enteropathogenic *E. coli* (EPEC), and *Citrobacter rodentium* (CR) that is secreted through the type III secretion system to host cells. The interaction between EspF and host proteins plays an important role in bacterial pathogenesis. EspF protein binds to host SNX9 and N-WASP proteins to promote the colonization of pathogenic bacteria in intestinal epithelial cells; combines with cytokeratin 18, actin, 14-3-3ζ, Arp2/3, profilin, and ZO-1 proteins to intervene in the redistribution of intermediate filaments, the rearrangement of actin, and the disruption of tight junctions; acts together with Abcf2 to boost host cell intrinsic apoptosis; and collaborates with Anxa6 protein to inhibit phagocytosis. The interaction between EspF and host proteins is key to the pathogenic mechanism of EHEC and EPEC. Here, we review how EspF protein functions through interactions with these 10 host proteins and contributes to the pathogenicity of EHEC/EPEC.

## Introduction

Over the last decade, the advancing field of cellular microbiology has provided a glimpse of the complex interactions between many bacteria and eukaryotic cells (Hartland and Richardson, 2016). One of the frontiers in this research is the battle between gram-negative enteric bacteria and certain host cells (Poulin and Chamaillard, 2017). Enteropathogenic *Escherichia coli* (EPEC), enterohemorrhagic *E. coli* (EHEC), and *Citrobacter rodentium* (CR) create unique histological lesions in intestinal epithelial cells and are called attaching and effacing pathogens (A/E pathogens) (Gaytán et al., 2016). EHEC and EPEC are a main cause of human disease (Wong et al., 2011). EPEC is the leading pathogen causing diarrhea in infants and young children (Donnenberg and Finlay, 2013), and acute renal failure and hemolytic uremic syndrome caused by EHEC are a source of mortality worldwide (Nguyen and Sperandio, 2012). CR is a mouse-restricted pathogen that shares several pathogenic mechanisms with EHEC and EPEC (Collins et al., 2014). These bacteria colonize the gut mucosa, mainly causing host diarrhea and colitis, even severe diarrhea, but their exact pathogenesis is still unknown.

EspF is one of the most important virulence factors of A/E pathogens, and its domain architecture and function have attracted considerable attention. It is injected into host cells through the type III secretion system (T3SS), targets host mitochondria and the nucleolus (Nougayrède and Donnenberg, 2004; Dean et al., 2010), disrupts tight junctions (Weflen et al., 2010), inhibits phagocytosis (Danika et al., 1999), induces characteristics of hemorrhagic enteritis such as the disappearance of microvilli in intestinal epithelial cells, cytoskeletal rearrangement, mitochondrial dysfunction, and apoptosis (Maddocks et al., 2013; Zhao et al., 2013). Mice infected with the *espF* mutant of CR show intestinal colonization reduction and colonic hyperplasia (Deng et al., 2004; Mundy et al., 2004), and rabbits infected with the EHEC *espF* mutant exhibit accumulation of polymorphonuclear leukocytes in colonic mucosa (Ritchie and Waldor, 2005). These studies indicate that EspF can promote pathogen colonization and modulate host inflammatory responses by suppressing or reducing host cytokines. Infection with the EPEC *espF* mutant fails to induce microvillous elongation, which occurs during normal infection, indicating a potential role for EspF in remodeling the brush border (Muza-Moons et al., 2004; Shaw et al., 2005). Moreover, the *espF* mutant impairs EPEC's ability to kill host cells, suggesting that EspF can induce host cell death (Crane et al., 2001). EspF has hence emerged as the “Swiss army knife” of EHEC/EPEC infection and pathopoiesis (Holmes et al., 2010). In addition, EspF can also exert biological effects by binding to host proteins. It cooperates with SNX9 and N-WASP proteins to promote the colonization of pathogens (Alto et al., 2007; Weflen et al., 2010); communicates with cytokeratin 18 (CK18), actin, 14-3-3ζ, Arp2/3, profilin, and zonula occludens-1 (ZO-1) proteins; disrupts tight junctions by redistributing intermediate filament protein CK18 and rearranging the actin cytoskeleton (Viswanathan et al., 2004a; Peralta-Ramírez et al., 2008); and interacts with Abcf2 to promote cell apoptosis (Nougayrède et al., 2007). Naturally, the capacity of EspF to affect host proteins is decisive in the pathogenesis of EHEC/EPEC.

The interaction of pathogenic effectors and host proteins has garnered increased attention (Kim et al., 2010). Apart from the domain architecture of EspF, its interaction with host proteins is also impressive. The challenge now is to identify host proteins that interact with EspF to decipher their effects on cellular physiology and provide molecular clues to EspF pathogenicity. In this review, we emphasize the cellular biological events produced by EspF-host-protein complexes, discuss recent observations of EspF and its binding proteins, describe some new insights into pathogen–host intercommunication, and discuss the molecular mechanisms of hemorrhagic enteritis and diarrhea caused by EHEC/EPEC infection.

## Domain architecture of the EspF protein

**Figure 1:**
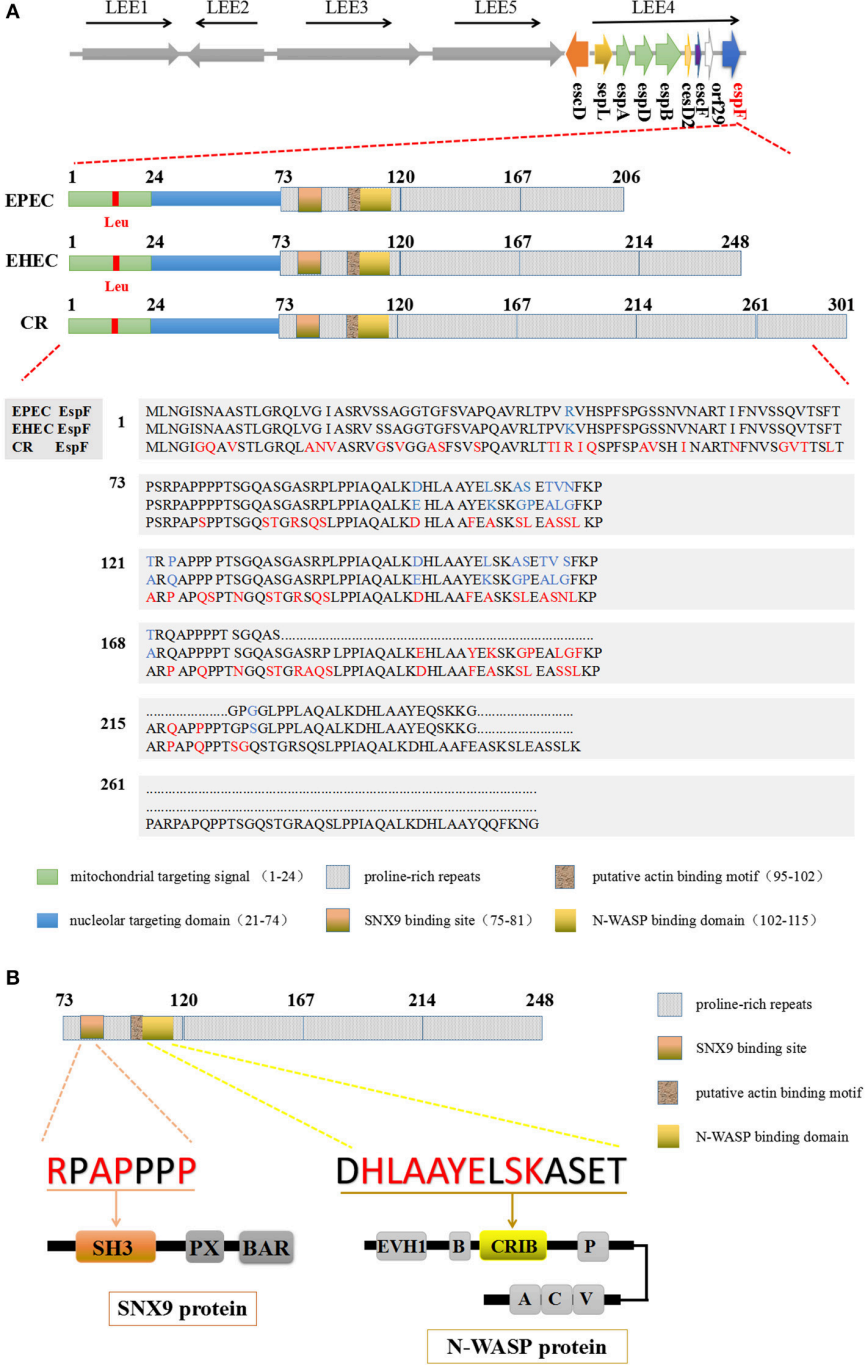
The domain architecture of the EspF protein. **(A)** EspF amino acid sequences and domain architecture diagrams of EPEC O127:H7 strain E2348/69, EHEC O157:H7 strain EDL933, and C. *rodentium* are shown. Differences in the size of their EspF proteins are caused by differences in the number of repeats of PRR in the C-terminal domain. **(B)** EHEC EspF protein C-terminal PRR repeats, including the SNX9 protein binding motif RxAPxxP, and the N-WASP protein binding sequence xHLAAYExSKxxxx.

A/E pathogens have a 35.5 kb LEE (locus of enterocyte effacement) pathogenicity island on their chromosomes (Gaytán et al., 2016). There are many virulence genes on the LEE island, including T3SS and six known effector proteins: Tir, Map, EspF, EspG, EspH, and EspZ (Wong et al., 2011; Gaytán et al., 2016). The *espF* gene is located on LEE4, the fourth operon of the LEE island. The N-terminal region (residues 1 to 73) of EspF is highly conserved, and secretory signal amino acid residues 1–20 of this region help EspF secrete from the bacteria and transport to host cells (Charpentier and Oswald, 2004). The mitochondrial targeting signal (residues 1–24) and the nucleolar targeting domain (residues 21–74) enable EspF to target the mitochondria and nucleolus of host cells (Holmes et al., 2010). The C-terminal region consists of 3–4 eukaryotic-like proline-rich repeats (PRRs), in which PRR1 contains a SH3 (src homology 3) binding motif PxxP, an effective Cdc42/Rac-interactive binding (CRIB) domain, and a possible actin binding domain (Holmes et al., 2010) (Figure 1A). The highly conserved RxAPxxP motif at residues 75–81 can bind specifically with the SH3 binding domain of the host cell SNX9 protein, and the EspF protein can also bind the N-WASP protein through the xHLAAYExSKxxxx sequence located at residues 102–115 (Figure 1B).

The *espF* gene sequences of EPEC and EHEC are up to 87% similar (Ugalde-Silva et al., 2016), while CR *espF* only has 67% similarity to that of EPEC and 65% to that of EHEC (Deng et al., 2001). CR has an *espF* gene of 906 bp with 5 PRRs and a corresponding 301 amino acid residues. EPEC has an *espF* gene of 621 bp, and the EspF protein has 206 amino acids with 3 PRRs, while the length of the EHEC *espF* gene is 747 bp with a corresponding amino acid size of 248 and 4 PRRs. Comparative sequence analysis shows that the EHEC EspF protein has 42 aa more than EPEC with 19 aa substitutions, while CR EspF protein has more than 50 aa variations from EHEC EspF (Figure 1A). The differences in EHEC and EPEC aa sequences are mainly concentrated in PRR1 and PRR2. These discrepancies lead to some differences in pathogenicity, such as in the reduction of epithelial resistance (Viswanathan et al., 2004b). Whole-genome sequencing of the CR virulent strain ICC168 has indicated that it shares a common host infection strategy with EHEC and EPEC (Petty et al., 2009), which makes CR an ideal model to study EPEC and EHEC infection *in vivo*.

EspF was first discovered in EPEC by McNamara and Donnenberg (1998). Since then, researchers have gradually increased our knowledge of EspF. Recently, Dean and Kenny have found that EPEC EspF can induce multinucleation and cell-cell internalization of intestinal epithelial cells accompanied by cell fusion events, which depend on its C-terminal proline repeat sequence (Dean and Kenny, 2013). This result reveals a new function of the C-terminal domain of the EspF protein. The domain architecture of EspF determines some of its interacting proteins, such as SNX9 and N-WASP, which determines its fate and plays an important role in its pathogenicity. It also important to determinewhether the differences in PRR repeats are just a result of DNA replication or if they have a host-specific role. At present, the structure of EspF protein is still under investigation, and we believe that there are more mysteries in EspF's structure that are worth exploring.

## Targeting of host cells

Currently, 10 proteins have been screened and verified to interact with EspF in host cells (Table 1), but how their interactions play a role in infection is poorly understood. We will analyze how EspF protein behaves as a versatile effector by interacting with host proteins, and discuss the potential effects in the pathogenesis of EHEC and EPEC.

**Table 1:** EspF host binding partners and their biological functions.

**Binding partner**	**Notes**	**The function of the protein itself ^a^**	**Biological effects of binding to EspF**
SNX9	Belongs to sorting nexin family	1. Interacts with adaptor protein 2, dynamin, tyrosine kinase non-receptor 2, Wiskott-Aldrich syndrome-like, and Arp3. 2. Participates in intracellular trafficking, including endocytosis, macropinocytosis, and F-actin nucleation.	1. Influences the regulation of clathrin-mediated endocytosis. 2. Mediates membrane remodeling. 3. Enhances the invasion of intestinal epithelial cells by EPEC.
N-WASP	Belongs to the Wiskott-Aldrich syndrome (WAS) family	1 1. Involved in transduction of signals from receptors on the cell surface to the actin cytoskeleton. 2. Associate with the small GTPase, Cdc42. 3. Regulates actin filament reorganization via its interaction with the Arp2/3 complex and mediate the formation of actin pedestals upon infection by pathogenic bacteria.	1. Mediates actin polymerization. 2. Induces Arp2/3-dependent actin assembly. 3.Mediates membrane remodeling.
Actin	Belongs to the actin family of proteins	1. Plays a role in cell motility, structure and integrity. 2. One of the most highly-conserved proteins known. 3. Is found in two main states: G-actin is the globular monomeric form, whereas F-actin forms helical polymers. Both G- and F-actin are intrinsically flexible structures.	1. Promotes pedestals maturation. 2. Disrupt paracellular permeability. 3. Mediated endocytosis of TJ proteins and may disrupt TJs.
Profilin	Small actin-binding proteins	1. Plays an important role in actin dynamics by regulating actin polymerization in response to extracellular signals. 2. Binds to actin and affects the structure of the cytoskeleton.	1. Promotes pedestals maturation. 2. Disrupt paracellular permeability. 3. Mediated endocytosis of TJ proteins and may disrupt TJs.
Arp2/3	Actin related protein 2/3	The Arp2/3 protein complex has been implicated in the control of actin polymerization in cells and has been conserved throughout evolution.	1. Cause the polymerization-depolymerization cycles of actin. 2. Promotes pedestals maturation. 3. Disrupts paracellular permeability. 4. Mediates endocytosis of TJ proteins and may disrupt TJs.
ZO-1	Zonula occludens-1, act as a tight junction adaptor protein	1. Act as a scaffold protein and regulate adherens junctions. 2. Interact with transmembrane proteins, cytosolic proteins, and F-actin, which are required for tight junction function. 3. Alternative splicing results in multiple transcript variants encoding different isoforms.	1. Causes polymerization-depolymerization cycles of actin. 2. Promotes pedestals maturation. 3. Disrupts paracellular permeability. 4. Mediates endocytosis of TJ proteins and may disrupt TJs.
Cytokeratin 18	Member of the intermediate filament gene family	1. Play a role in filament reorganization. 2. Cadherin binding involved in cell-cell adhesion	1. Changes the architecture of the intermediate filament network. 2. May disrupt TJs.
14-3-3ζ	A member of the 14-3-3 protein family	1. Interacts with IRS1 protein, suggesting a role in regulating insulin sensitivity. 2. Acts as a suppressor of apoptosis and has a central role in tumor genesis and progression. 3. Involved in the regulation of cellular actin structures through the maintenance of phosphorylated-cofilin levels.	1. Modulates the solubility and distribution of cytokeratin 18. 2. Changes the architecture of the intermediate filament network. 3. May disrupt TJs.
Abcf2	Belongs to the ABC protein superfamily	1. Be characterized as the product of an iron-inhibited transcribed gene. 2. Act as a cytoprotective, anti-apoptotic factor.	Facilitates host cell death.
Anxa6	Belongs to a family of calcium dependent membrane and phospholipid binding proteins	1. Annexin VI has been implicated in mediating the endosome aggregation and vesicle fusion in secreting epithelia during exocytosis. 2. Alternatively spliced transcript variants have been described. 3. May associate with CD21. 4. May regulate the release of Ca(2+) from intracellular stores.	1. May rearrange cytoskeleton. 2. May inhibit phagocytosis. 3. May downregulate EGFR.

a *The function of the protein itself comes from UniProtKB/Swiss-Prot Function*.

### Targeting mitochondria

The targeting of EspF protein to host cells is mainly determined by its N-terminal domain. EPEC EspF targets mitochondria through its mitochondrial targeting domain, and accelerates the targeting through the mitochondrial membrane protein Tom20, which it may interact with (Muto et al., 2001; Nagai et al., 2005). This results in the destruction of mitochondrial membrane potential (MMP), the release of cytochrome *c* into the cytoplasm, the cleavage of caspases 9 and 3, and the initiation of the mitochondrial apoptosis pathway, eventually leading to cell death (Nougayrède and Donnenberg, 2004). The transportation of EspF to mitochondria is crucial in the EHEC/EPEC infection process. Nagai et al. have replaced the leucine at position 16 with glutamate, and this alteration completely abolishes the mitochondrial targeting of EspF and consequently affects the mitochondrial apoptosis pathway; this observation confirms that the 16th Leu plays a decisive role in the mitochondrial targeting of EspF (Nagai et al., 2005).

### Targeting the nucleolus

EspF was the first bacterial protein recognized to target the nucleolus. Paul et al. found that EPEC utilize mitochondria to control the timing of and extent to which EspF targets the nucleolus. In the early stage of infection, EspF accumulates in mitochondria and causes the loss of MMP, which determines when EspF is available for targeting the nucleolus. In the later stage of infection, EspF targets the nucleolus through its N-terminal domain, leading to the relocalization of nucleolin to the cytoplasm and a reduction in the level of the ribosomal protein RPL9 (Dean et al., 2010). This nucleolar targeting of EspF is strictly controlled by EPEC's modulation of host mitochondria (Dean et al., 2010), thus we speculate that the process of targeting mitochondria must occur before targeting the nucleolus. Although the role of EspF targeting the nucleolus in EPEC disease is unclear, the revelation that bacterial pathogens use host organelles for spatiotemporal control over its effector proteins provides us with new insights into bacterial effector targeting.

A proteomic analysis has shown that the amount of many ribosomal proteins decreases after EPEC infection (Hardwidge et al., 2004), and pre-rRNA processing is blocked in host cells expressing EspF, which requires the EspF nucleolar-targeting domain (Dean et al., 2010). EPEC may destroy the ribosome biosynthesis process by adjusting ribosomal protein levels after a period of infection. We hypothesize that there exists some cooperation between EspF and ribosomal proteins, and that EspF may attenuate ribosome processing by targeting the endoplasmic reticulum. Although its role in disease is obscure, it may help pathogens escape host cell defense responses by reducing the synthesis of ribosomal proteins in long-term infections.

## Promotion of bacterial colonization

The specific interaction of EPEC EspF and SNX9 protein in intestinal epithelial cells was first discovered by Marche et al. using immunoprecipitation and confocal microscopy in 2006; the proteins colocalize in HeLa cells (Marchès et al., 2006). Subsequently, Alto et al. confirmed that EPEC EspF not only interacts with SNX9 protein but also with neuronal Wiskott-Aldrich syndrome protein (N-WASP) (Alto et al., 2007).

The SNX9 protein contains three conserved regions: SH3, PX, and BAR (Figure 1B). SH3 consists of 50–60 aa and is responsible for the recognition of cellular signal proteins that are rich in proline-producing PxxP modules (such as the Src and Abl tyrosine kinase protein families), and for mediating protein interactions (Aitio et al., 2010; Bendris and Schmid, 2017). The SNX9 protein is an intracellular membrane regulator that can cause the formation of membranous tubules, induce cell membrane remodeling, and promote bacterial invasion (Weflen et al., 2010). The SNX9 and SNX18 proteins form a heterodimer in the membrane that activates the GTPase domain of dynamin and interacts with N-WASP (Park et al., 2010). EPEC EspF forms a protein complex with SNX9 and N-WASP and actives an endogenous SNX9/N-WASP signaling pathways to regulate diverse eukaryotic trafficking events (Alto et al., 2007; Weflen et al., 2009; Garber et al., 2018).

The C-terminal BAR domain of SNX9 is a membrane-interacting promoter that can sense changes in membrane curvature and induce membrane tubularization (Chen et al., 2013). SNX9 targets clathrin-coated pits (CCPs) that are rich in bis- and trisphosphorylated phosphatidylinositol molecules; this targeting specificity allows the regulation of SNX9 and its binding proteins, which include N-WASP and dynamin, during clathrin-mediated endocytosis (CME) (Shin et al., 2008). SNX9 also binds to clathrin and adaptor protein 2 (AP2) through a motif in the low complexity (LC) domain to further strengthen its localization in CCP (Lundmark and Carlsson, 2002). The combination of specificity and high affinity between EspF and SNX9 remodels the plasma membrane, leading to plasma membrane deformation, and these membrane remodeling events are directly related to N-WASP/Arp2/3-mediated actin nucleation (Alto et al., 2007).

**Figure 2:**
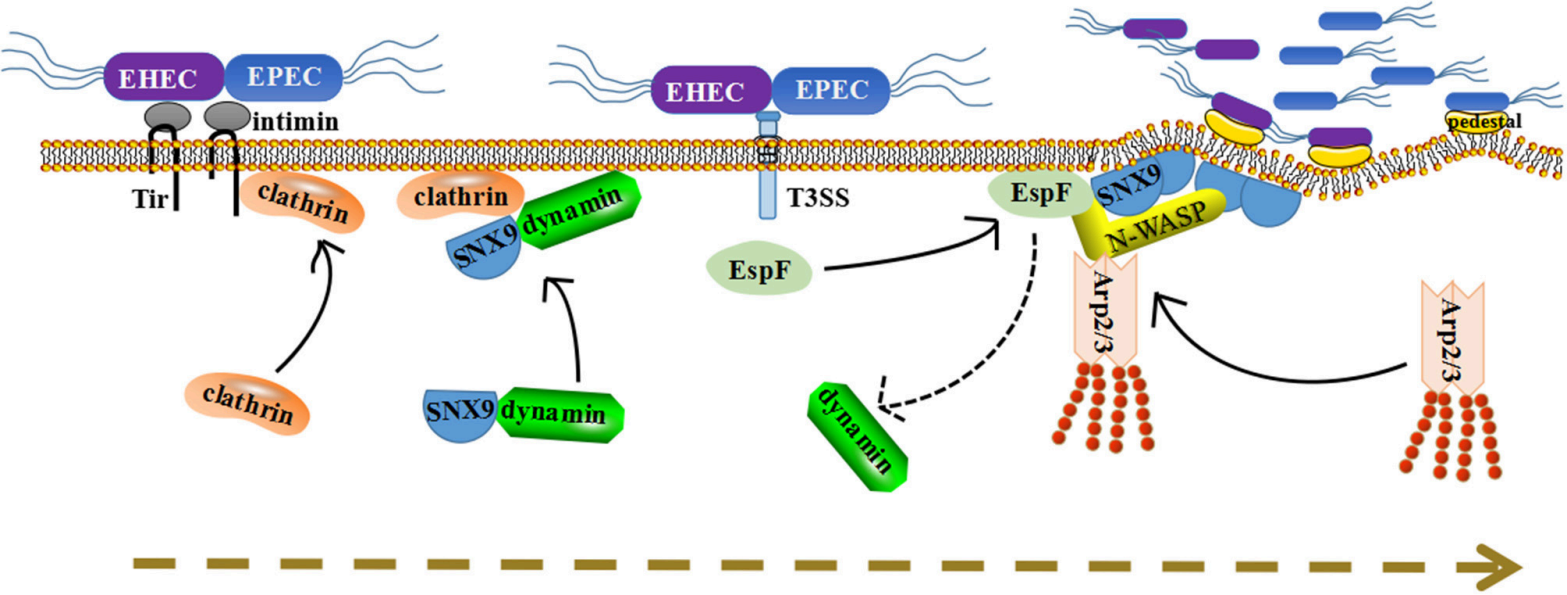
A dynamic model for how EspF protein potentially promotes the colonization of bacteria in host cells through protein interactions. First, Tir inserts into the plasma membrane and recruits clathrin to accumulate at the point of pathogen attachment. Second, under normal circumstances, SNX9 will recruit its partner dynamin to membrane areas. Once EspF comes, it binds to SNX9 protein in competition with dynamin, and their solid interaction induces SNX9 oligomerization and increases membrane deformation activity. Third, SNX9 interacts with N-WASP, as well as with EspF, to form a complex and trigger Arp2/3-dependent polymerization of branched-chain actin filaments. Thus, we propose that their interaction facilitates the colonization of pathogenic bacteria step-by-step: membrane deformation, actin polymerization, pedestal formation, and colonization promotion.

EPEC EspF promotes the colonization and invasion of pathogenic bacteria to intestinal epithelial cells by relying on the interaction with SNX9 (Weflen et al., 2010). The activation of N-WASP and SNX9 by EspF may be a pathogenic strategy to mimic the SNX9/N-WASP signaling complex in the natural host. We propose a model in which EspF utilizes multiple steps to promote the colonization and invasion of pathogens: recruitment to the plasma membrane, membrane deformation, actin polymerization, and pedestal formation (Figure 2).

First, after EPEC/EHEC contacts the host, the translocated intimin receptor (Tir) is secreted into the cytoplasm of epithelial cells (Campellone, 2010), then Tir inserts into the plasma membrane and interacts with intimin on the surface of EPEC/EHEC, providing a foothold for further adhesion of pathogenic bacteria onto epithelial cells (Kenny et al., 1997).

Second, Tir recruits clathrin and causes it to accumulate at the point of pathogen attachment. EPEC/EHEC adhesion leads to a series of changes in the plasma membrane, such as PIP accumulation, aggregation of phosphorylated tyrosine membrane receptor Tir, and even the formation of a curved membrane surface (Touz et al., 2004; Sason et al., 2009; Weflen et al., 2010). These provide a favorable environment for the recruitment of SNX9 proteins. Under normal circumstances, SNX9 will recruit its partner dynamin to these membrane areas. After recruitment, EspF interacts with the SH3 domain of SNX9 protein, and this interaction requires at least two SNX9 binding sites (Weflen et al., 2010). The 3–4 binding sites in EspF may facilitate the binding of EspF to the SNX9 SH3 domain in competition with dynamin, and further consolidate its interaction with SNX9. Multiple SNX9 binding domains allow EspF to bind 3–4 molecules of SNX9, subsequently inducing SNX9 oligomerization and increasing membrane deformation activity.

Third, membrane remodeling events are associated with N-WASP/Arp2/3-mediated actin nucleation. SNX9 combines with N-WASP through its SH3 domain, thus stimulating N-WASP to trigger Arp2/3-dependent polymerization of branched-chain actin filaments (Alto et al., 2007). EspF has 3-4 N-WASP binding sites, allowing it to recruit and interact with N-WASP to initiate actin fiber branching and assembly through the Arp2/3 complex (Alto et al., 2007; Weflen et al., 2010). Thus, we propose that EspF activates SNX9 and N-WASP through spatial coordination and regulates its peripheral proteins to cause membrane deformation, actin polymerization, and pedestal formation, thereby facilitating the colonization of pathogenic bacteria.

Although the formation of pedestals is of significance for the colonization of pathogens, its specific functional mechanism is still undetermined. The adhesion of pathogenic bacteria to epithelial cells may make the bacteria more resistant to fluid-mediated separation during diarrhea. In any case, EspF clearly forms a protein complex with SNX9 and N-WASP, and this plays a pivotal role in promoting bacterial colonization.

## Destruction of tight junctions

One of the characteristics of EPEC/EHEC infection is increased permeability of solutes through intestinal epithelial cells (Viswanathan et al., 2004b). Intestinal epithelial cells adhere to adjacent cells through an adhesive complex that includes tight junctions (TJs), adherens junctions, and desmosomes (Singh et al., 2018). Upon infection, the distribution of tight junction proteins is changed and the tight junction structure and barrier function are disrupted. EPEC EspF plays a pivotal role in the destruction of TJs and the augmentation of membrane permeability, resulting in the loss of transepithelial electrical resistance and the relocation of the tight junction-associated protein occluding (Zhang et al., 2010), but the specific mechanism has not been defined. We speculate that EspF protein may destroy TJs gradually by recruiting a series of proteins such as actin, profilin, N-WASP, ZO-1, etc., and combining different proteins in different biological processes.

Tight junctions are complex structures that are key in establishing polarity and barrier functions, and they are located in the most apical region of epithelial and endothelial cell junction complexes (Van Itallie and Anderson, 2014). Tight junctions, like a fence, limit the diffusion of lipids and intimal proteins between the apical and basolateral membranes to establish polarity and barrier function (Turner et al., 2014). In addition, they act as physical barriers that regulate the paracellular transportation of water, ions, solutes, and immune cells (Pawłowska and Sobieszczanska, 2017). The transmembrane proteins occludin, ZO-1, and claudin are well-known tight junction functional proteins (Runkle and Mu, 2013). Occludin and claudins directly regulate the permeability of uncharged and charged molecules, respectively. ZO-1 serves as a link between the cytoskeleton and TJs. ZO-1, claudin, and occludin interact with actin via different domains, and their interactions contribute to the molecular linkage between the cytoskeleton and tight junction complexes (Van Itallie et al., 2009; Günzel and Fromm, 2012; Runkle and Mu, 2013; Krug et al., 2014; Zihni et al., 2016).

EspF of rabbit enteropathogenic *E. coli* (E22) interacts with actin and profilin, and this interaction takes place throughout the infection process (Peralta-Ramírez et al., 2008). Profilin is a small-molecule protein that binds to actin monomers and then delivers them to the fast-growing end of actin filaments (Pantaloni and Carlier, 1993; Witke, 2004). EspF may be involved in the regulation of actin polymerization as a nucleation promoting factor through direct interaction with actin or indirect interactions between profilin and actin.

EspF of E22 interacts with N-WASP, Arp2/3, ZO-1, and ZO-2 directly or indirectly within 2 h post-infection (Peralta-Ramírez et al., 2008). The cooperation of EspF with these proteins is not a collection of independent events but a series of consecutive related events. EspF binds to N-WASP and Arp2/3, inducing actin polymerization and pedestal formation. EspF of E22 interacts with actin and immobilizes it, allowing the recruitment of junction proteins into the pedestal, resulting in the redistribution of tight junction proteins, the maturation of the actin-rich pedestals, and the disruption of paracellular permeability (Peralta-Ramírez et al., 2008). EspF of E22 can also bind to ZO-1 and ZO-2 scaffold proteins, sequestering actin and profilin, inducing local actin depolymerization, and resulting in the imbalance of polymerization-depolymerization cycles (Peralta-Ramírez et al., 2008). Studies have shown that depolymerization of actin disrupts tight junctions through caveolin-mediated endocytosis of occluding (Shen and Turner, 2005).

During EPEC infection, EspF can also interact with cytokeratin 18 (CK18) and 14-3-3ζ to form a complex that increases the solubility of CK18 and alters the distribution of intermediate filaments, resulting in the decomposition of the intermediate filament network (Viswanathan et al., 2004a). However, the role of this event in the pathogenicity of EPEC is still undetermined. EPEC has been demonstrated to enhance myosin light chain (MLC) phosphorylation (Yuhan et al., 1997), and EspF may combine with calmodulin through 14-3-3ζ to activate and phosphorylate MLC, which disturbs the tight junction barrier process.

Recent studies have shown that EPEC EspF depletes junction proteins through transcriptional and post-transcriptional mechanisms and interacts with ZO-1 to regulate tight junction assembly and disassembly, thereby affecting the integrity of the barrier and disrupting tight junctions (Singh et al., 2018). EPEC EspF can also promote the endocytosis of Crumbs3 (Crb3) by binding to SNX9, which disrupts the polarity of intestinal epithelial cells, destroys tight junctions, changes the absorption of ions and solutes by membrane transporters, and promotes EPEC-associated diarrhea (Tapia et al., 2017).

**Figure 3:**
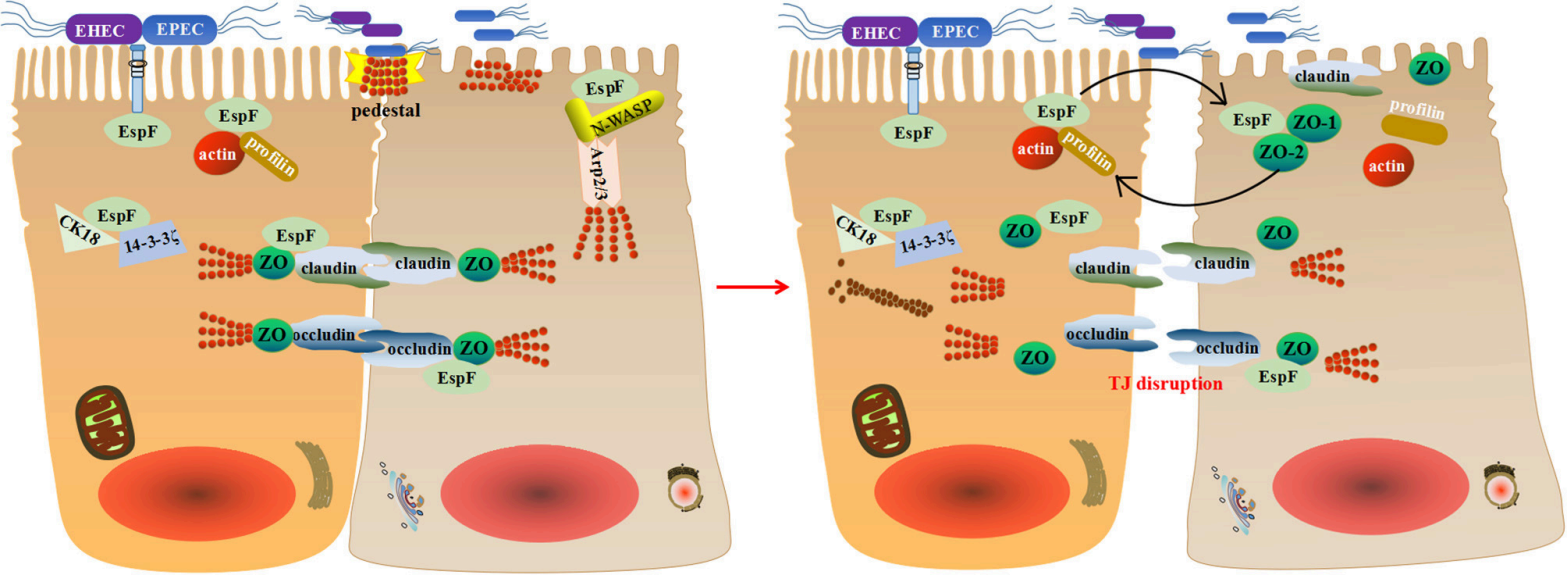
A schematic model for how EspF potentially disrupts tight junctions. EspF interacts with CK18 and 14-3-3ζ to redistribute intermediate filaments and combines with N-WASP, Arp2/3, actin, profilin, and ZO-1 to recruit junctional proteins to the pedestal. This results in the redistribution of tight junction proteins, depolymerization of actin, and interruption of tight junctions.

Thus, we propose that EspF may disrupt TJs through a multipronged strategy during EPEC/EHEC infection: it interacts with actin and profilin at the pedestal to immobilize actin, recruits junction proteins to the pedestal, promotes the maturation of the pedestal, and disrupts paracellular permeability. Then tight junction proteins occludin, claudin, and ZO-1 redistribute, resulting in transepithelial resistance loss. EspF then binds to the scaffold proteins ZO-1 and ZO-2, causing actin depolymerization, resulting in an unbalanced state of polymerization-depolymerization, and thus TJ breakdown (Figure 3). Although the specific mechanism of EspF-induced TJ disruption remains mysterious, its interactions with N-WASP, Arp2/3, actin, profilin, ZO-1, CK18, and 14-3-3ζ provide clues.

## Inhibition of phagocytosis

The internalization process is detrimental to EHEC/EPEC, and recruiting macrophages are often an effective strategy for host cells to prevent and eliminate infections (Sarantis and Grinstein, 2012). Correspondingly, pathogens also have coping strategies. EspF protein is a highly evolved coping strategy.

There are two steps involved in the internalization process of EHEC/EPEC, the transportation process of M cells and then the phagocytosis by macrophages (Martinez-Argudo et al., 2007). M cells are specialized epithelial cells that are distinguished from other epithelial cells by their high transport capacity. When pathogens invade, M cells transport them to downstream immune cells, such as macrophages, to clear them (Brayden et al., 2005; Mabbott et al., 2013).

Although M cells have high transport capacity, studies have shown that their capacity to transport EPEC is lower than that of *Salmonella*. The deletion of T3SS or EspF protein significantly increases the translocation rate, and a phosphatidyl inositol 3-kinase (PI3K) inhibitor can decrease the translocation rate of EPEC strains with loss of T3SS function (Martinez-Argudo et al., 2007).

EPEC mediates antiphagocytosis by inhibiting the PI3K pathway, and the EspF protein inhibits the phagocytosis of EPEC by J774.A1 macrophages via a PI3K-dependent pathway, which depends on its N-terminal domain, playing a crucial part in the anti-phagocytosis process (Celli et al., 2001; Quitard et al., 2006). The role of EspF in regulating EPEC transportation by M cells appears to be similar to its antiphagocytic effect in macrophages.

Poirier et al. confirmed that EHEC O157:H7 survives after infecting human macrophages; although the macrophages try to clear the pathogens, after 24 h of infection, some infected macrophages hold larger numbers of bacteria than at early infection points, indicating that the bacteria not only survive but replicate inside macrophages (Poirier et al., 2008). However, our understanding of the anti-phagocytotic mechanisms of EHEC is very poor.

Recently, we screened and verified that Annexin A6 (Anxa6) interacts with EHEC O157:H7 EspF using Bimolecular Fluorescence Complementation (BiFC) technology for the first time (Hua et al., 2018). Anxa6 belongs to a highly conserved protein family characterized by calcium-dependent binding to phospholipids. As a multifunctional scaffold protein, Anxa6 is involved in many biological processes including cell proliferation, survival, differentiation, and inflammation (Grewal et al., 2017). It can interact with actin, leading to the formation of membrane-cytoskeletal complexes, which may affect actin dynamics by recruiting signaling proteins and forming complex protein interaction networks, thereby remodeling the actin cytoskeleton (Hayes et al., 2004; Mishra et al., 2011; Grewal et al., 2017). The significance of actin for phagocytosis has been well-documented (Castellano et al., 2001; Smythe and Ayscough, 2006; Carlsson, 2017), and the actin-binding molecule profilin is also recruited to FCγR-mediated phagocytic cups (Coppolino et al., 2001).

The actin cytoskeleton plays a critical role in regulating the epidermal growth factor receptor (EGFR) cycle and controlling endocytosis and degradation of EGFR (Da Costa et al., 2003; Smythe and Ayscough, 2006). Anxa6 can interact with the actin cytoskeleton and may lead endocytic vesicles to multivesicular bodies. Anxa6 can also bind to p120GAP and PKCα, thus negatively controlling the EGFR/Ras pathway (Grewal and Enrich, 2009).

EHEC/EPEC infection can regulate the host cytoskeleton by activating PKCα and recruiting PKC to form adhesion pedestals through functionally intact lipid rafts (Crane and Oh, 1997; Shen-Tu et al., 2014), but the mechanism is undetermined. The activation of PKC is controlled by the PI-3 (phosphatidyl inositol 3)/AKT signaling pathway, and EPEC relies on this pathway to escape the phagocytosis of host cell macrophages (Celli et al., 2001; Shen-Tu et al., 2014). EPEC participates in the activation of EGFR and causes the phosphorylation of EGFR, which promotes host cell survival in early infection, but EspF accelerates the loss of EGFR in late infection leading to a dramatic increase in host cell death (Roxas et al., 2007, 2014).

**Figure 4:**
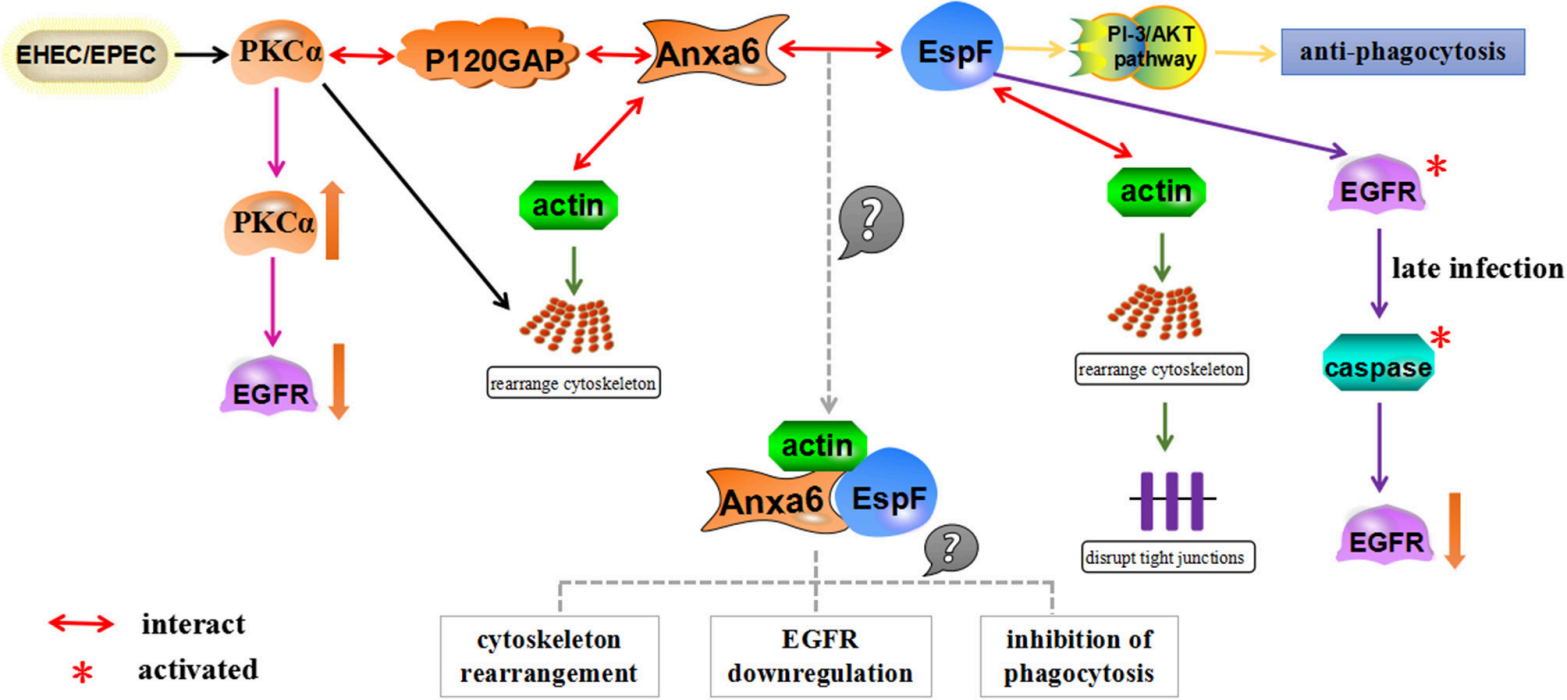
A schematic model for how EspF and Anxa6 protein potentially mediate anti-phagocytosis. Anxa6 collaborates with actin to cause cytoskeletal rearrangement, forms complexes with PKCα and P120GAP, and downregulates EGFR levels. Similarly, EspF combines with actin to regulate the rearrangement of the actin cytoskeleton in host cells, exerts anti-phagocytosis through the PI-3/AKT pathway, and leads to a decrease in EGFR levels through activation of caspase in late infection. EspF and Anxa6 may form complexes with actin, which act together to mediate cytoskeleton relocation, EGFR downregulation, and anti-phagocytosis.

The detection of Anxa6 protein bound to EspF may provide us with a new mechanism of EspF-mediated anti-phagocytosis: EspF forms a complex with Anxa6 and actin after EPEC/EHEC infects host cells, which regulates the rearrangement of the actin cytoskeleton; reorganization of the cytoskeleton modulates the PI-3/AKT pathway, triggers the activation of PKCα and the phosphorylation of EGFR, and induces the down-regulation of EGFR. As the complex activates the PI-3/AKT signaling pathway, it restricts phagocytosis and assists in the survival of pathogenic bacteria in macrophages (Figure 4).

We suspect that Anxa6 may be an essential bridge protein for EspF to develop anti-phagocytosis and down-regulate cellular EGFR levels, and we are conducting further research to test this.

## Regulation of apoptosis

During infection of intestinal epithelial cells, the surface properties of EPEC/EHEC induce exogenous apoptotic pathways (Abul-Milh et al., 2001), whereas T3SS effectors such as EspF, Map, and cycle inhibitory factor (Cif) trigger intrinsic apoptotic pathways (Wong et al., 2011).

By destroying the mitochondrial membrane potential, EspF initiates the intrinsic apoptotic pathway, leading to the release of cytochrome *c*, the cleavage of caspases 3 and 9, and eventually cell apoptosis (Nougayrède and Donnenberg, 2004). Our previous research confirmed that the N-terminal region of the EHEC O157:H7 EspF protein causes cell apoptosis (Zhao et al., 2013), and an N-terminal domain-deleted strain reduces the mitochondrial binding affinity of EHEC (Wang et al., 2017).

Up to now, only one EspF-interacting protein, Abcf2, has been shown to be involved in apoptosis (Nougayrède et al., 2007). Abcf2 belongs to the ATP-binding cassette (ABC) transporter superfamily and is a cytoprotective anti-apoptotic factor (Ando-Akatsuka et al., 2012; Bao et al., 2017). After infection of EPEC, the host cell Abcf2 protein level is decreased, and the levels of caspase 9 and caspase 3 in Abcf2 gene-silenced cells are reduced, which depend on EspF, indicating that EspF binds to Abcf2 and inhibits its anti-apoptotic effect, thereby inducing or promoting cell apoptosis (Nougayrède et al., 2007). This interesting work highlights the usefulness of identifying interacting proteins in eukaryotic cell biology, because it suggests that the relatively unknown Abcf2 protein is an anti-apoptotic factor.

Although apoptotic cells issue “find-me” and “eat-me” signals (Davidovich et al., 2014), in the early stages of EHEC/EPEC infection, some inflammatory factor signaling pathways may be triggered, such as NF-κB (Pallett et al., 2014; Yen et al., 2016). EHEC/EPEC applies some mechanism to restrain the early inflammatory response to obtain a longer survival period before the host's overall immune response is induced (Sharma et al., 2006; Ruchaud-Sparagano et al., 2007; Nobe et al., 2009); for example, EPEC can deliver effector Nlec to suppress innate immune responses by inhibiting NF-κB and MAPK activation (Pearson et al., 2011; Sham et al., 2011). In 2013, Professor Donnenberg of the University of Maryland proposed a hypothesis that A/E *E. coli* chronic infection can promote the occurrence of human rectal cancer (Maddocks et al., 2013). A/E *E. coli* infects intestinal epithelial cells and injects virulence proteins that causes DNA damage in the host cells, which increases cancer risk along with the Toll-like receptor signaling pathway, the NF-κB pathway, and other cellular inflammatory pathways (Vogelmann and Amieva, 2007; Maddocks et al., 2009; Kipanyula et al., 2013). Further, EspF decreases host cell DNA mismatch repair (MMR) levels, which can then lead to mutations in the *Apc* gene (Maddocks et al., 2013). Destruction of the MMR system leads to an increase in the mutation frequency of tumor suppressor genes *Apc* and *p53*, which are considered to be the most mutagenic genes in colorectal cancer (Smith et al., 2002; Maddocks et al., 2013). This discovery directly confirmed Donnenberg's hypothesis. However, whether all A/E bacterial chronic infections can lead to the occurrence of colorectal cancer, and the specific mechanism, remains to be further investigated.

Apoptosis is a multifactor-mediated event. Increased bacterial colonization, disruption of tight junctions, and inhibition of phagocytosis initiated by EspF binding to host proteins may indirectly lead to eventual cell apoptosis. Apoptosis is also the beginning of the body's immune response, and the process from apoptosis to the generation of inflammation is complicated. We believe that EspF has some other interaction partners that, in addition to co-promoting apoptosis, may also trigger cell inflammatory signaling pathways, leading to cell death. This is worthy of further study.

## Conclusions

The human body has a variety of innate defense mechanisms to resist the invasion of microorganisms. Host proteins play a decisive role in the immune response, phagocytosis, prevention of adhesion and colonization, and other processes. Nevertheless, many pathogens are equipped with highly evolved infectious strategies, for example secreting “smart” effectors like EspF, which can not only inject into the host cell, but also interact with some host proteins and take advantage of their function to mediate virulence, promote bacterial survival, and destroy host cells.

**Figure 5:**
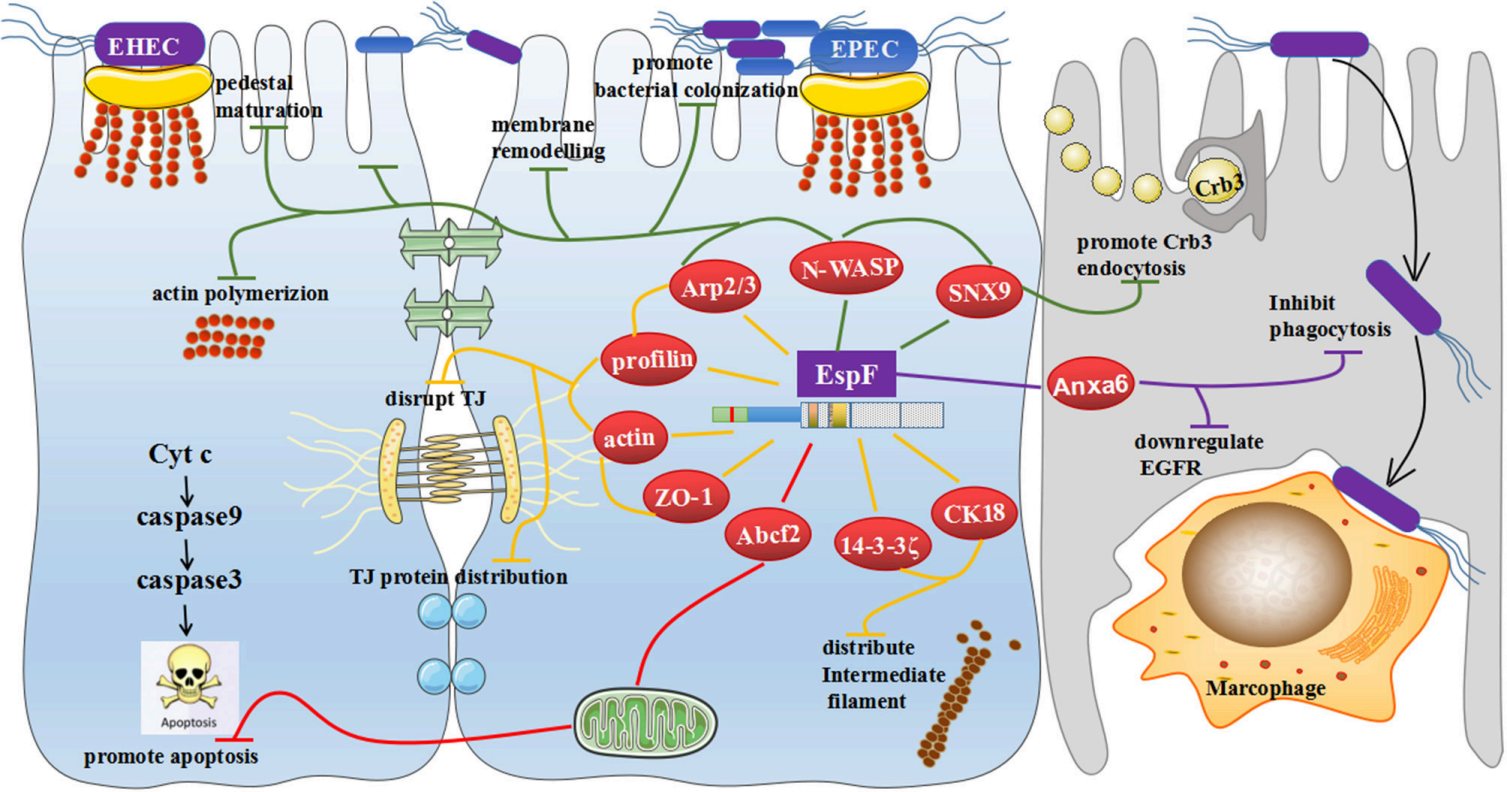
General view of the biological effects mediated by EspF binding to host proteins. EspF interacts with SNX9 to promote endocytosis of the Crb3 protein; combines with SNX9, N-WASP, and Arp2/3 proteins to maturate pedestals; regulates actin polymerization to induce remodeling of cell membranes and potentially promote colonization of pathogenic bacteria; cooperates with Arp2/3, profilin, actin, and ZO-1 to redistribute actin and potentially disrupt the TJs; interacts with 14-3-3ζ and CK18 to redistribute intermediate filaments, which may also promote the destruction of TJs; interacts with Abcf2, which may mediate apoptosis through the mitochondrial pathway; and interacts with Anxa6, which may downregulate EGFR levels and inhibit phagocytosis.

This review has focused on the clever cooperation between EspF and host proteins: it interacts with SNX9 and promotes endocytosis of Crb3 protein; combines with SNX9, N-WASP, and Arp2/3 proteins to promote pedestal maturation, regulate actin polymerization, induce cell membrane remodeling, and potentially further the colonization of pathogenic bacteria; cooperates with Arp2/3, profilin, actin, and ZO-1 to cause actin redistribution and potentially disrupt TJs; interacts with 14-3-3ζ and CK18 to redistribute intermediate filaments and may also promote tight junction destruction; binds to Abcf2 and facilitates host cell apoptosis; and interacts with Anxa6, which may downregulate EGFR levels and mediate anti-phagocytosis (Figure 5).

EHEC and EPEC infections are characterized by the rapid onset of diarrhea (Viswanathan et al., 2009). EspF's role in causing diarrhea is the ability to disrupt TJs, but the precise mechanism of EspF's binding to host proteins to cause diarrhea has not been defined. In our previous research, we screened AQP7P2, a type of water channel protein that interacts with EspF (Hua et al., 2018). Their interaction may induce diarrhea by changing the activity of water molecule transport. EspF may also mediate the development of diarrhea by interacting with different host proteins. The interaction of EspF with Abcf2 protein results in apoptosis, which may be associated with inflammation caused by EPEC, but this has not been confirmed.

Although the interaction between EspF and host proteins has been widely investigated, many problems remain. Since most of the data related to EspF are from research on EPEC rather than EHEC or CR, we may have neglected some other interaction effects, or the interaction effects of different A/E pathogens may be different. It is also possible that EspF binds with other virulence proteins to mediate the interaction with host proteins, and these have yet to be studied.

Current research has mainly focused on the interaction between EspF and intestinal epithelial cells. In the future, we need to learn more about the interaction of EspF with immune cells and their role in the pathogenesis of EHEC/EPEC, and explore how EspF influences or participates in innate and adaptive immune responses through interactions with host proteins. The discovery of Anxa6 protein provides us with a new foothold, as we speculate that it may be an key bridge protein for EspF to inhibit phagocytosis and down-regulate EGFR levels. EspF binding to Anxa6 may trigger the PI-3/AKT signaling pathway, promote anti-phagocytosis, activate cellular PKCα protein, negatively regulate EGFR signaling, and exacerbate host cell death. These areas are primed for further research.

## Author contributions

CW, YH designed and wrote the paper. KY collected the data. Thank all the authors' contribution to the manuscript.

### Conflict of interest statement

The authors declare that the research was conducted in the absence of any commercial or financial relationships that could be construed as a potential conflict of interest.
